# The Impact of Social Media Addiction on Vaccine Hesitancy Among Nursing Students: Testing an Empirical Model

**DOI:** 10.1002/nop2.70460

**Published:** 2026-03-16

**Authors:** Bethany Nichol, Goran Erfani, Jemma McCready, Charlotte Gordon, John Unsworth, Michelle Croston, Dania Comparcini, Valentina Simonetti, Giancarlo Cicolini, Kristina Mikkonen, Jeremia Keisala, Marco Tomietto

**Affiliations:** ^1^ School of Healthcare and Nursing Sciences, Faculty of Health and Wellbeing Northumbria University Newcastle upon Tyne UK; ^2^ School of Communities and Education, Faculty of Health and Wellbeing Northumbria University Newcastle upon Tyne UK; ^3^ Policy Research Unit for Behavioural and Social Sciences, Population and Health Sciences Institute Newcastle University Newcastle upon Tyne UK; ^4^ Manchester University NHS Foundation Trust Manchester UK; ^5^ Interdisciplinary Department of Medicine University of Bari “Aldo Moro” Bari Italy; ^6^ Department of Innovative Technologies in Medicine and Dentistry “Gabriele D'Annunzio” University of Chieti Chieti Italy; ^7^ Research Unit of Health Science and Technology University of Oulu Oulu Finland; ^8^ Medical Research Center Oulu Oulu University Hospital and University of Oulu Oulu Finland; ^9^ Research Unit of Health Science and Technology, Faculty of Medicine University of Oulu Oulu Finland

**Keywords:** information, nursing students, public health, social media, vaccine hesitancy

## Abstract

**Introduction:**

Vaccine hesitancy is relevant among nursing students, as they move across healthcare and university settings. Given the opportunity of social media to disseminate misleading information to users and the exposure of the digital natives, social media addiction (SMA) is considered a predictor of vaccine hesitancy, although to date no research within this specific population exists.

**Purpose:**

To investigate the role of SMA on vaccine hesitancy in nursing students.

**Design:**

Cross‐sectional.

**Method:**

Structural Equation Modelling was adopted to test the study's aim. The VAX scale was used among 227 nursing students in the UK regarding COVID‐19 and Influenza vaccines during the period from March to July 2023. The Bergen Social Media Addiction scale was adopted to measure SMA.

**Results:**

Statistically significant results were found between SMA and mistrust of vaccine benefits. No significant correlations were found between SMA and concerns about unforeseen future effects or a preference for natural immunity. Regarding concerns about commercial profiteering, there was a significant negative correlation for the COVID‐19 vaccine.

**Conclusion:**

Mistrust in institutions and scepticism towards vaccine benefits and commercial interests are recurring issues tied to social media. Targeting popular social media platforms for tailored vaccination campaigns is recommended to promote vaccine acceptance among nursing students.

**Implications for the Profession and/or Patient Care:**

Providing accurate and accessible vaccine information on social media platforms, promoting media literacy and engaging with individuals to address their concerns are key to promoting vaccination among nursing students.

**Impact:**

This study provides new strategies for tailoring vaccination campaigns and policies. Public health efforts to combat vaccine hesitancy should consider the role of social media and work to provide accurate and balanced information to the public.

**Reporting Method:**

The Strengthening the Reporting of Observational Studies in Epidemiology (STROBE) guidelines were adopted.

**Patient or Public Contribution:**

No patient or public contribution.

## 1 Introduction

Vaccine hesitancy, defined as the delay in refusal or acceptance of vaccination, despite the availability of safe vaccination services (MacDonald 2015), is identified as one of the top ten threats to global health (World Health Organisation 2019). Worryingly, healthcare workers (HCWs) show higher vaccine hesitancy towards COVID‐19 vaccination than the general population (McCready et al. 2023). Nurses in particular are the least likely to have received the seasonal influenza and the COVID‐19 vaccine (Dror et al. 2020; McCready et al. 2023), despite being the most represented occupation in healthcare settings (OECD 2021).

## 2 Background

Vaccine hesitancy is a relevant phenomenon among nursing students, who divide 50% of their time between university and healthcare settings (EUR‐Lex 2019) and are subsequently at a greater risk of both contracting communicable diseases and spreading them to patients and peers through interaction within healthcare settings, an active social life and interactions within the students' community (Asad et al. 2020). Moreover, addressing vaccine hesitancy in nursing students can be seen as a long‐term investment to promote vaccination in nurses, as the clinical learning environment provides an opportunity to address any normative misconceptions (Jaffe et al. 2022) before students become full‐time practicing nurses, particularly as previous receipt of vaccination is often reported as the strongest predictor of influenza and COVID‐19 vaccination (Cheung et al. 2017; Luo et al. 2021). Furthermore, given that perception of others' behaviour is a unique predictor of vaccination (Jaffe et al. 2022), addressing vaccine hesitancy in this population may help to drive bottom‐up change in vaccination within healthcare settings, given that nursing students represent the new generation of nurses. Despite this, among nursing students, intention to receive and acceptance of COVID‐19 vaccination averages 44% (Patelarou et al. 2021) and 60% (Geng et al. 2022) respectively, and acceptance of influenza vaccination in the last year ranges from 15% (Cheung et al. 2017) to 33% (Salem et al. 2019), indicating a need to increase vaccination.

An investigation into the reasons for vaccine hesitancy in nursing students will allow for targeted interventions throughout their education and clinical learning experiences (McCready et al. 2023), with the aim to address hesitancy before they are qualified and ultimately increase COVID‐19 and influenza vaccination rates among nurses. The sparse available literature concerning nursing students indicates key barriers to include fear of side effects (Belingheri et al. 2021; Manning et al. 2021; Salem et al. 2019; Yeşiltepe et al. 2021) and safety of the vaccine (Fontenot et al. 2021; Manning et al. 2021; Salem et al. 2019; Zhou et al. 2021). These aspects resonate with the four major factors defining vaccine hesitancy, namely mistrust of vaccine benefit, worries about unforeseen future effects, concerns about commercial profiteering and preference for natural immunity (McCready et al. 2024; Martin and Petrie 2017). Furthermore, these aspects are strongly linked to the spread of mis‐ and disinformation about vaccines. Especially during the pandemic a concerning amount of fake news was disseminated, defined as ‘fabricated information that imitates news media content in form but not in organizational process or intent, which overlaps with other information disorders, such as misinformation—false or misleading information—and disinformation, which is false information that is deliberately disseminated to deceive people’ (Lazer et al. 2018). This news largely spread through social media, which is largely native to the student population.

Social media allows for the dissemination of unregulated and often unreliable information, resulting in increased vaccine hesitancy (Shakeel et al. 2022), leading to a reduced vaccination rate (Burki 2019). For example, a survey conducted across 17 countries, including the UK, found use of all social media platforms predicted conspiracy beliefs around COVID‐19, aside from Twitter (‘X’) which exerted a negative effect (Theocharis et al. 2021), which in turn predicts negative attitudes towards vaccination (Rosli et al. 2022). Social media use for COVID‐19 vaccine information has also been identified as a facilitator of vaccination in university students in China, demonstrating an important role of the type and content of social media consumption in its prediction of vaccine hesitancy (Mo et al. 2021). Social media has also been reported to negatively impact vaccination in healthcare professionals: an umbrella review found that reliance on unreliable sources of information, such as social media, predicted vaccine hesitancy in comparison with retrieving information from reliable sources, such as national news (McCready et al. 2023). Less research has been conducted concerning healthcare students, although existing literature indicates a similar pattern (McCready et al. 2023). In addition, vaccine hesitancy is also a significant predictor of the use of social media to discourage others around vaccination (Chadwick et al. 2021), potentially creating a feedback loop.

Further, problematic social media use has been described as an addiction (Andreassen et al. 2016), exacerbated by the COVID‐19 pandemic (Zhao and Zhou 2021). Although the only behavioural addiction associated with technology in the Diagnostic and Statistical Manual of Mental Disorders (DSM‐5) (American Psychiatric Association and DSM‐5 Task Force 2013) is internet gaming disorder, available literature indicates addiction to media and particularly social media meets criteria for classification as a distinct diagnosis (Pinna et al. 2015). Namely, social media addiction (SMA) is described to possess six core elements (Andreassen et al. 2017; Griffiths 2005); a preoccupation with social media (salience), use of social media to reduce negative emotions (mood modification), the need to use social media more and more to receive the same dopamine response (tolerance), feelings of distress when taken away from or unable to use social media (withdrawal), failed attempts to stop or cut down social media use (relapse), and an impairment to daily living in sacrifice to engagement with social media (functional impairment). SMA has been linked to reduced mental health (Brailovskaia et al. 2021; Keles et al. 2020) and wellbeing (Duradoni et al. 2020) alongside outcomes such as depression and anxiety (Brailovskaia et al. 2021; Keles et al. 2020). More recently, SMA was also reported as a significant predictor of vaccine hesitancy (Ahorsu et al. 2022; Erinç et al. 2022), mediated by fear of and perceived risk of COVID‐19 (Ahorsu et al. 2022). This phenomenon is particularly important among those born after 1997 (generation Z) who identify themselves as ‘digital natives’ and are competent in and enthusiastic towards digital solutions (Hammarén et al. 2024) such as social media. Other generations have a different relationship and confidence with digital solutions and social media. Specifically, people born between 1946 and 1964 are defined as baby boomers, those born between 1965 and 1980 as generation X, those born between 1981 and 1996 as generation Y, and individuals from all three generations show a lower engagement with social media compared to generation Z (Schmitt and Lancaster 2019).

In summary, there is a need for an investigation into the predictors of influenza and COVID‐19 vaccine hesitancy within nursing students. Also, few studies have investigated the role of social media use on vaccine hesitancy or uptake in healthcare students, and none for nursing students or SMA specifically. Thus, the current study aims to investigate the role of SMA on the factors of vaccine hesitancy in nursing students, enabling novel insights into this phenomenon.

## 3 Method

### 3.1 Aim

This study aims to evaluate the association between SMA and the factors of vaccine hesitancy (mistrust of vaccine benefit, worries about unforeseen future effects, concerns about commercial profiteering, preference for natural immunity) among nursing students, by considering Flu and COVID‐19 vaccines.

### 3.2 Design

This was a cross‐sectional survey study.

### 3.3 Hypotheses

*SMA significantly and positively relates to the factor ‘mistrust of vaccine benefit’*.

*SMA significantly and positively relates to the factor ‘worries about unforeseen future effects’*.

*SMA significantly and positively relates to the factor ‘concerns about commercial profiteering’*.

*SMA significantly and positively relates to the factor ‘preference for natural immunity’*.

**FIGURE 1:**
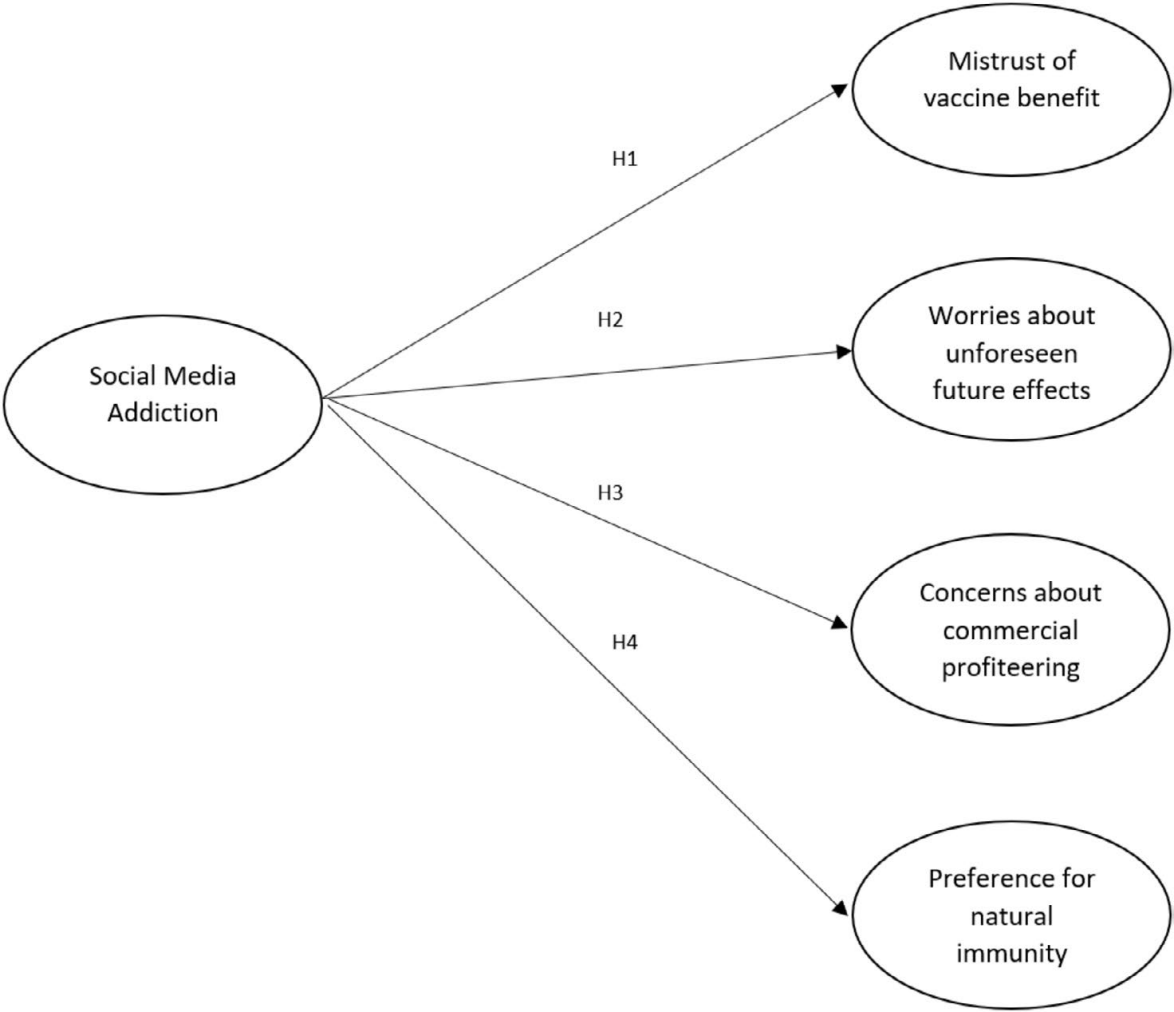
Hypothetical model and study hypotheses for Influenza and COVID‐19 vaccines.

These hypotheses were tested for both Flu and COVID‐19 vaccines and supported by the evidence that outlines how social media usage and addiction may affect the beliefs regarding the vaccines' effectiveness (H1 and H4) (Rosli et al. 2022), long‐term safety (H2) (Tomietto et al. 2022), and trust in the institutions (H3) (Dopelt et al. 2023). Figure 1 represents the model tested and the study hypotheses.

### 3.4 Measures

To measure vaccine hesitancy, the VAX scale was adopted. The scale consists of 12 items rated on a Likert scale of agreement, ranging from one (strongly disagree) to seven (strongly agree) (Huza 2020; Martin and Petrie 2017). The scale was used with respect to both COVID‐19 and Influenza vaccines, as these are the two priority vaccines to address to enhance vaccination coverage among nursing students. The scale covers four factors of vaccine hesitancy: mistrust of vaccine benefit (three items) defined as the belief that vaccines are not effective or beneficial in preventing diseases; worries about unforeseen future effects (three items) defined as the concerns that vaccines may have long‐term negative health effects that are currently unknown; concerns about commercial profiteering (three items) defined as the belief that vaccines are promoted primarily for the financial benefit of pharmaceutical companies rather than for public health; and preference for natural immunity (three items) defined as the belief that natural immunity (gained through contracting and recovering from a disease) is superior to vaccine‐induced immunity (Martin and Petrie 2017). To measure all items in the same direction, three items related to ‘mistrust of vaccine benefit’ were reversed, including ‘I feel safe after being vaccinated’, ‘I can rely on vaccines to stop serious infectious diseases’ and ‘I feel protected after getting vaccinated’. Lower mean scores reflected a more positive attitude towards the vaccine. Responses were mandatory for all items of the VAX scale.

The Bergen Social Media Addiction Scale (BSMAS) (Andreassen et al. 2016) was used to measure SMA. The scale includes six items rated on a Likert scale of usage of social media from one (very rarely) to five (very often). Higher scores reflect a more addictive attitude towards using social media.

Socio‐demographic, work‐related (i.e., work or placement in a COVID‐19 area), and health‐related (COVID‐19 infection exposure) data were also collected to describe the sample, based on the variables affecting vaccine hesitancy as identified in the literature (McCready et al. 2023; Dopelt et al. 2023).

### 3.5 Participants and Sample Size

The inclusion criterion for participant recruitment in this study was being an undergraduate or postgraduate nursing student in the UK.

Structural Equation Modelling was adopted to test the study's hypotheses. Therefore, a participant to item ratio ranging from 10:1 to 20:1 was recommended (Kline 2015). In this study, the model to test the hypotheses included 12 items from the VAX scale and six items from the BSMAS. Hence, a sample size ranging from 180 to 360 participants was considered adequate to test the hypotheses.

### 3.6 Data Collection

Data were collected between March and July 2023, by involving nursing students via an online survey developed on the JISC Online Surveys platform and disseminated nationwide in the UK via formal and informal networks involving academic contacts and student associations. A convenience sampling strategy was adopted, combined with a snowball sampling approach.

### 3.7 Data Analysis

Descriptive statistics were used to describe the sample. Categorical variables were presented as frequencies (percentages) and continuous variables as means, standard deviation, median, minimum and maximum.

Multivariate normality is an assumption to run a Structural Equation Model (SEM). To test multivariate normality, Mahalanobis distances and their *p*‐values of chi‐square distribution, with 30 degrees of freedom, were calculated to identify multivariate outliers among the responses related to VAX scale for COVID‐19 and Influenza and the BSMAS. Outliers were identified as 12 cases with Mahalanobis distance values exceeding the critical *χ* ^2^ value of 58.77 at the 0.001 level. By keeping multivariate outliers, the data distribution did not verify multivariate normality (Mardia's kurtosis coefficient = 1130.88); by deleting them, Mardia's kurtosis coefficient was found to be 913.47, below the critical value of 960, and multivariate normality was verified (Mikkonen et al. 2022). In detail, 12 multivariate outliers were excluded from the final sample.

The SEM model has been tested by calculating the fit indices for the SEM model shown in Figure 1, representing the study hypotheses. The fit indices are considered acceptable for a RMSEA (root mean square error of approximation) and SRMR (standardised root mean residual) < 0.08, and based on a CFI (comparative fit index) and TLI (Tucker‐Lewis index) > 0.90. Additionally, the Coefficient of Determination (CD) was calculated to represent the variance explained by the models (Byrne 2016; Kline 2015). A multi‐stage approach was adopted to test the hypothetical model: if a parameter of the hypothetical model was not significant, it was removed from the initial model and the fit indices re‐tested in the new model until a satisfactory fit was achieved.

Missing data analysis was not required because the VAX scale was mandatory and no missing data were identified in the BSMAS in this sample.

Data analysis was performed by using SPSS and AMOS v28 (IBM Corp 2021).

### 3.8 Validity and Reliability

To establish the content validity of the scale, the scales were preliminarily sent to a panel of eight healthcare experts. The items were assessed for clarity and relevance to calculate the scale Content Validity Index (S‐CVI). Several studies (Polit and Beck 2006; Yusoff 2019) previously recommended that the cut‐off value of the content validity index (CVI) should be set at 0.83 at the scale level (S‐CVI). In this study, the relevance and clarity of both scales were satisfactory: for the VAX scale were respectively 0.86 and 0.88, while for the BSMAS were respectively 0.83 and 0.89.

The scales' reliability was tested by calculating Cronbach's alpha for each factor of the scales. Cronbach's alpha values > 0.90 are considered excellent, values > 0.70 and < 0.90 good, values > 0.60 and < 0.70 acceptable and values < 0.60 non‐acceptable (DeVellis and Thorpe 2021).

In this study, Cronbach's alpha coefficients for the VAX scale for COVID‐19 vaccine ranged between 0.83 and 0.97 among factors. Cronbach's alpha of the VAX scale for Flu ranged from 0.75 to 0.92 among factors, and for the BSMAS (six items) was 0.85. All scales demonstrated satisfactory reliability.

A Confirmatory Factor Analysis was performed for each scale to test construct validity, the fit indices considered for SEM modelling were considered and specifically, RMSEA and SRMR < 0.08 and CFI and TLI > 0.90 (Kline 2015).

In this study, the VAX scale for COVID‐19 and Influenza showed respectively RMSEA = 0.057 (90% CI = 0.035–0.077), SRMR = 0.039, CFI = 0.987, TLI = 0.982 and RMSEA = 0.052 (90% CI = 0.029–0.073), SRMR = 0.043, CFI = 0.983, TLI = 0.976. The BSMAS demonstrated RMSEA = 0.042 (90% CI = 0.001–0.098), SRMR = 0.025, CFI = 0.995, TLI = 0.985. All scales achieved optimal construct validity. Both scales are available in the public domain and validated in several studies internationally. In detail, the VAX scale was already adopted and validated in Europe, South America and South Africa, confirming the four‐factor structure and optimal validity and reliability (Padmanabhanunni et al. 2023). The BSMAS scale has been validated in several European countries, China and the United States, confirming its psychometric characteristics (Brailovskaia and Margraf 2024). In this study, both scales confirmed their validity and reliability in their original form.

### 3.9 Sensitivity Analysis

Given that vaccine hesitancy and social media addiction may show a generational pattern (Tomietto et al. 2022; Brailovskaia et al. 2021), the mean scores of the VAX scale and its factors and the BSMAS scale were compared across generations using ANOVA.

### 3.10 Ethical Considerations

This study was approved by Northumbria University (Reference Number: 2948 27/02/23). All information and data gathered during the study were stored and analysed in line with the General Data Protection Regulation GDPR (European Parliament 2018) and the UK Data Protection Act (2018). Data were stored within the Northumbria University cloud system, using a secure password‐protected log‐in process. Survey completion was anonymous and confidential. Informed consent was obtained by each participant before starting the survey.

## 4 Results

### 4.1 Participants Characteristics

In total, 239 students in nursing completed both the VAX scale and the BSMAS. The final sample size was 227 after removing the multivariate outliers (*n* = 12). Participants were on average 26.63 years old (SD = 8.25, median = 23, min = 18, max = 59) and 89.4% of them were female. The majority of the sample belonged to generation Z (*n* = 142, 62.6%) while the remaining to generation Y (*n* = 72; 31.7%) and a minority to generation X and Baby Boomers (in total *n* = 13; 5.7%). The average number of clinical placement experiences in the last 12 months was 3.22 (SD = 1.01, median = 3, min = 0, max = 6). Regarding the level of academic study, most participants reported BSc (Hons) 212 (93.4%) and the rest MSc 15 (6.6%). Only 24 (10.6%) participants reported interruption in their study. Reported fields of nursing were as follows: Adult 166 (73.1%), Child 30 (13.2%), Mental Health 25 (11.0%), and Learning Disability 6 (2.6%). The 79.7% of the participants experienced clinical placements in a COVID‐19 area, and 194 (85.5%) reported having been affected by COVID‐19. The results show that 200 (88.1%) participants were vaccinated against COVID‐19 whilst only 93 (41%) were vaccinated against Flu. In terms of social media app usage, the highest levels were reported for Instagram (Mean = 3.17, SD = 0.98) and then WhatsApp (Mean = 3.12, SD = 0.89). The lowest levels of social media usage were reported for LinkedIn (Mean = 1.17, SD = 043) and then Reddit (Mean = 1.26, SD = 0.68). Table 1 reports the characteristics of the sample.

**TABLE 1:** The socio‐demographic characteristics of the sample (*n* = 227).

Variables	Mean	SD
Age	26.63	8.25

	Frequency (*n*)	Percentage (%)
Gender		
Male	22	9.7
Female	203	89.4
Others	2	0.9
Level of study		
BSc (Hons)	212	93.4
MSc	15	6.6
Field of nursing		
Adult	166	73.1
Child	30	13.2
Mental health	25	11.0
Learning disability	6	2.6
Year of programme (BSc)		
Year 1	23	10.1
Year 2	154	72.6
Year 3	35	16.5
Year of programme (MSc)		
Year 1	11	73.3
Year 2	4	26.7
Interruptions in study^a^	24	10.6
Clinical placements in a COVID‐19 area^a^	181	79.7
Vaccinated against Influenza^a^	93	41.0
Been affected by Covid^a^	194	85.5
Vaccinated against Covid^a^	200	88.1

Social media apps usage	Mean	SD
Facebook	2.76	1.00
YouTube	2.60	0.94
WhatsApp	3.12	0.89
Instagram	3.17	0.98
TikTok	2.91	1.27
Snapchat	2.76	1.26
Pinterest	1.62	0.79
Reddit	1.26	0.68
LinkedIn	1.17	0.43
Twitter	1.80	0.99

*Note:* Participants were on average 26.63 years old (SD = 8.25) and the average number of clinical placement experiences in the last 12 months was 3.22 (SD = 1.01).

^a^ Reflects the number and percentage of participants answering ‘yes’ to this question.

The overall score for vaccine hesitancy was 4.03 (SD = 0.76) for the COVID‐19 vaccine and 4.02 (SD = 0.71) for the Influenza vaccine, while the SMA score was 2.61 (SD = 0.89) in the overall sample. Mistrust of vaccine benefit reported the highest score for both COVID‐19 and Influenza vaccines, respectively: 4.96 (SD = 1.73) and 5.47 (SD = 1.31). Table 2 reports the detailed scores of the VAX scale for each factor and item regarding COVID‐19 and Influenza vaccines. Table 3 describes the Bergen Social Media Addiction scale's scores.

**TABLE 2:** Vaccination hesitancy of nursing students (*n* = 227).

Factor/items statements	COVID‐19		Influenza	
	Mean	SD	Mean	SD
Mistrust of vaccine benefit	4.96	1.73	5.47	1.31
I feel safe after being vaccinated^a^	4.99	1.83	5.41	1.49
I can rely on vaccines to prevent serious infectious diseases^a^	4.99	1.74	5.50	1.43
I feel protected after getting vaccinated^a^	4.91	1.78	5.50	1.30
Worries about unforeseen future effects	4.60	1.34	4.42	1.25
Although most vaccines appear to be safe, there may be problems that we have not yet discovered	5.31	1.35	5.41	1.27
Vaccines can cause unforeseen problems in children	4.07	1.52	3.81	1.47
I worry about the unknown effects of vaccines in the future	4.44	1.80	4.04	1.84
Concerns about commercial profiteering	3.19	1.57	3.02	1.39
Vaccines make a lot of money for pharmaceutical companies, but do not do much for regular people	3.59	1.74	3.53	1.74
Organisations promote vaccination for financial gain, not for people's health	3.37	1.75	3.26	1.60
Vaccination programs are a big fraud	2.59	1.56	2.26	1.33
Preference for natural immunity	3.38	1.60	3.19	1.36
Natural immunity lasts longer than a vaccination	3.54	1.66	3.41	1.47
Natural exposure to viruses and germs gives the safest protection	3.40	1.70	3.33	1.55
Natural exposure to diseases is safer than being exposed through vaccination	3.20	1.70	2.84	1.52
Overall vaccination hesitancy	4.03	0.76	4.02	0.71

^a^ The scores for these positively worded items were reversed prior to calculating the mean scores.

**TABLE 3:** SMA of nursing students (*n* = 227).

Item no.	Statements	Mean	SD
1	… spent a lot of time thinking about social media or planning how to use it	2.68	1.16
2	… felt the urge to use social media more and more	2.87	1.17
3	… used social media in order to forget about personal problems	2.96	1.29
4	… have tried to decrease the use of social media without success	2.74	1.18
5	… became restless if you have no access to social media	2.37	1.23
6	… *used social media so much that it has had a negative impact on your job/studies*	2.03	1.11
Overall social media addiction		2.61	0.89

### 4.2 Hypotheses' Testing

The parameters of the hypothetical model were tested on both Influenza and COVID‐19 vaccines. Specifically, significant correlations were identified between social media addiction and vaccine benefit mistrust for both Influenza (0.19) and COVID‐19 (0.25) vaccines, both of which were statistically significant (respectively *p* < 0.01 and *p* < 0.001). However, no statistically significant correlations were found between social media addiction and concerns about unforeseen future effects or a preference for natural immunity.

Regarding concerns about commercial profiteering, no significant correlation was found with social media addiction for the Influenza vaccine, but a statistically significant and negative correlation for the COVID‐19 vaccine (−0.15, *p* < 0.001) was observed (Model 1, Table 5). The overall models did not meet the criteria for satisfactory fit indices. Specifically, the model for the Influenza vaccine yielded RMSEA = 0.110, SRMR = 0.219, CFI = 0.840 and TLI = 0.813, while the model for the COVID‐19 vaccine resulted in RMSEA = 0.129, SRMR = 0.288, CFI = 0.850 and TLI = 0.825. Both models explained 86.3% of the variance (CD = 0.863) (Model 1, Tables 4 and 5).

**TABLE 4:** Influenza model's parameters estimation and statistical tests (*n* = 227).

Model	Outcome variable	Explanatory variable	Parameter	Standard error	*z*‐test	*p*
1	Mistrust of vaccine benefit	SMA	0.19	0.07	2.61	**0.009**
	Worries about unforeseen future effects	SMA	−0.04	0.08	−0.45	0.650
	Concerns about commercial profiteering	SMA	−0.04	0.08	−0.54	0.591
	Preference for natural immunity	SMA	−0.03	0.08	−0.40	0.687
2	Mistrust of vaccine benefit	SMA	0.18	0.07	2.53	**0.011**

	Fit indices					
	Chi‐square/*p*	RMSEA	SRMR	CFI	TLI	CD
1	486.93/< 0.001	0.110	0.219	0.840	0.813	0.863
2	76.20/< 0.001	0.092	0.056	0.952	0.934	0.864

*Note:* Bold values represent statistical significance.

**TABLE 5:** COVID‐19 model's parameters estimation and statistical tests (*n* = 227).

Model	Outcome variable	Explanatory variable	Parameter	Standard error	*z*‐test	*p*
1	Mistrust of vaccine benefit	SMA	0.25	0.07	3.49	**0.000**
	Worries about unforeseen future effects	SMA	−0.03	0.08	−0.41	0.678
	Concerns about commercial profiteering	SMA	−0.15	0.08	−2.02	**0.044**
	Preference for natural immunity	SMA	−0.07	0.07	−0.97	0.331
2	Mistrust of vaccine benefit	SMA	0.24	0.07	3.38	**0.001**
	Concerns about commercial profiteering	SMA	−0.14	0.07	−1.87	0.061
3	Mistrust of vaccine benefit	SMA	0.22	0.07	3.17	**0.002**

	Fit indices					
	Chi‐square/*p*	RMSEA	SRMR	CFI	TLI	CD
1	622.82/< 0.001	0.129	0.288	0.850	0.825	0.863
2	234.85/< 0.001	0.125	0.203	0.911	0.887	0.863
3	67.51/< 0.001	0.084	0.053	0.970	0.958	0.864

*Note:* Bold values represent statistical significance.

However, after removing the non‐statistically significant correlations, both models demonstrated satisfactory fit indices. In particular, the model correlating social media addiction with mistrust of vaccine benefit for the Influenza vaccine showed a similar and statistically significant parameter (0.18; *p* = 0.011) and satisfactory fit indices: RMSEA = 0.092, SRMR = 0.056, CFI = 0.952, TLI = 0.934 and CD = 0.864 (Model 2, Table 4). When worries about unforeseen future effects and preferences for natural immunity were excluded from the model regarding COVID‐19 vaccine, the correlation between social media addiction and concerns about commercial profiteering lost statistical significance (−0.14; *p* = 0.061), confirming unsatisfactory fit indices (Model 2, Table 5). As a result, the COVID‐19 model was tested solely on the correlation between social media addiction and mistrust of vaccine benefit, confirming a similar and statistically significant parameter (0.22, *p* = 0.002). Under this revised approach, the fit indices were satisfactory, with RMSEA = 0.084, SRMR = 0.053, CFI = 0.970, TLI = 0.958 and CD = 0.864 (Model 3, Table 5).

### 4.3 Sensitivity Analysis

The comparison between the SMA score and the VAX scores for the overall scale and its factors was performed using ANOVA. In detail, the SMA score was significantly higher in generation Z compared to generation Y and X/BB (respectively 2.83 ± 0.84, 2.27 ± 0.85 and 2.12 ± 0.93; *F* = 12.575, *p* < 0.001). The overall VAX score did not show any statistical difference across generations; however, the factors ‘mistrust of vaccine hesitancy’ and ‘concerns of commercial profiteering’ showed significantly higher scores respectively in generation Z and generation Y (Table 6).

**TABLE 6:** Sensitivity analysis. ANOVA comparing generations, SMA and VAX scores.

Variables	Generations	*N*	Mean	SD	*F*	*p*
SMA	Z	142	2.83	0.84	12.575	**0.000**
	Y	72	2.27	0.85		
	X/BB	13	2.12	0.93		
Overall vaccine hesitancy (VAX scale score)	Z	142	4.00	0.76	1.067	0.346
	Y	72	4.13	0.78		
	X/BB	13	3.84	0.63		
Mistrust of vaccine benefit	Z	142	5.19	1.57	4.018	**0.019**
	Y	72	4.49	1.95		
	X/BB	13	5.08	1.73		
Worries about unforeseen future effects	Z	142	4.55	1.25	1.321	0.269
	Y	72	4.78	1.43		
	X/BB	13	4.21	1.78		
Concerns about commercial profiteering	Z	142	2.99	1.47	3.885	**0.022**
	Y	72	3.61	1.70		
	X/BB	13	2.97	1.46		
Preference for natural immunity	Z	142	3.28	1.58	1.309	0.272
	Y	72	3.63	1.69		
	X/BB	13	3.10	1.33		

*Note:* Bold values represent statistical significance.

## 5 Discussion

This study explored the role of Social Media Addiction on vaccine hesitancy and highlighted how social media affect the perception of mistrust of vaccine benefit for both Influenza and COVID‐19 vaccines. Descriptive statistics indicated that the largest driver of vaccine hesitancy using the VAX scale was mistrust around the Influenza and COVID‐19 vaccines. When hypotheses were tested using a hypothetical model, statistically significant positive correlations were found between SMA and mistrust of vaccine benefits for both Influenza and COVID‐19 vaccines, but not concerns about unforeseen future effects or a preference for natural immunity. Between SMA and concerns about commercial profiteering, there was no significant correlation with the Influenza vaccine, but there was an initial significant negative correlation for the COVID‐19 vaccine. However, the final model did not confirm this correlation. Hence, mistrust of vaccine benefits remains the main factor of vaccine hesitancy affected by SMA.

Vaccine hesitancy among healthcare students is a widely debated topic and some patterns have already been highlighted by previous research. For example, female students were found to be generally more hesitant (Jennings et al. 2021) and Black/African American students displayed more hesitancy than those from other minorities (Wang and Liu 2022). A major role in vaccine hesitancy is related to the lack of information about the vaccine benefits and its side effects. Social media use for vaccine‐related information had mixed effects and much depends on the individual exposure to specific media and social media channels (Erfani et al. 2024; Krishnan et al. 2021). This is consistent with previous research which disclosed the complex interaction between social media addiction, perceived risk of being vaccinated, and vaccine hesitancy (Ahorsu et al. 2022). The overexposure to vaccine‐related content on social media and mis‐ and disinformation on these topics can influence individuals' beliefs and contribute to vaccine hesitancy (Ahorsu et al. 2022). However, some factors such as the risk perception of getting infected (Ahorsu et al. 2022), the critical attitude in evaluating information (Arede et al. 2019), past experiences, and educational background (McCready et al. 2023) can mitigate the impact of social media on vaccine hesitancy.

Mistrust of institutions and criticism towards commercial profiteering and governmental decisions are recurrent factors related to social media use (Boucher et al. 2021). While it is expected that nursing students have a higher critical attitude in evaluated health‐related information, their exposure to social media should be considered and it is necessary to reinforce their trust of vaccine benefit to foster their vaccination acceptance. Nursing students are predominantly part of generation Z (born after 1997), which is a native digital generation with an extensive integration of social media in everyday life (Schmitt and Lancaster 2019). While this generation has been found to be the most prone to vaccination in nursing (Tomietto et al. 2022), the impact of social media on the specific factors of vaccine hesitancy was not yet explored. This study shows that generation Z is more susceptible to social media addiction who also show higher scores regarding mistrust of vaccine hesitancy. This finding is consistent with the findings from previous studies where mistrust of institutions and concerns regarding the healthcare system's decisions regarding vaccination is a major factor of vaccine hesitancy among nursing students (Dopelt et al. 2023). It also indicates that social media can play a relevant role in addressing this component of vaccine hesitancy. In this way, this study fills this gap by showing that, to further improve nursing students' vaccination acceptance, social media channels should promote trust in the vaccine's benefit and in the academic and governmental institutions. This is even more important among nursing students as they are socially active and, most of all, their academic life crosses both the University environment and the clinical setting, with clear implications for their own individual safety and that of their patients. In this perspective, vaccination should be perceived by nursing students as a component of their future professional role and responsibility, and as a matter of patient safety (Wilson et al. 2020; Cheung et al. 2017). According to our results, the main social media channels to be targeted should be Instagram, WhatsApp, TikTok, Facebook and Snapchat. In particular, Snapchat was found in other studies to be associated with higher vaccine hesitancy (Erfani et al. 2024). Other authors found that users of Instagram, Snapchat, TikTok and YouTube express higher hesitancy towards COVID‐19 vaccine (Jennings et al. 2021). These results support tailored vaccination campaigns through the key social media platforms nursing students adopt the most and by tailoring the information to deliver through those channels.

### 5.1 Limitations

This study employed a cross‐sectional design, and inference on causal pathways should be considered with caution. Furthermore, the nursing student population includes students from different age categories, and a generational pattern might be further explored in testing the model. Most of the sample in this study included participants belonging to generation Z; hence, it was not possible to test the model on different generations, as the sample size was not adequate to run a multigroup model. The sample size can be considered representative of the nursing students in the UK. However, a larger sample may cover wider geographical areas, including students from different social and ethnic backgrounds. The data collection was disseminated via an online platform, and this approach may have excluded students with limited access to digital devices. Further research could explore this model on the general population from different social and ethnic backgrounds and identify generational patterns to better tailor the social media channels and the core messages to deliver to foster vaccination acceptance.

### 5.2 Implications for Policy and Practice

The findings from the current study inform future interventions to address vaccine hesitancy in nursing students whilst avoiding the important ethical issues associated with mandatory vaccination. To address the potential negative impact of social media on vaccine hesitancy, targeted public health campaigns and interventions are needed at the public health level by academic and governmental institutions. These may involve providing accurate and accessible vaccine information on social media platforms, promoting media literacy, and engaging with individuals to address their concerns and provide credible information. It is necessary to promote awareness and health literacy in the use of unregulated media and social media channels, as well as to work with the regulators on specific policies to ensure reliable information on these platforms. Public health efforts to combat vaccine hesitancy should consider the role of social media and work to provide accurate and balanced information to the public.

## 6 Conclusion

Vaccine hesitancy is a major concern in healthcare settings, and promoting vaccination acceptance among nursing students is key due to their exposure to clinical practice and their professional role development. This study explored vaccine hesitancy with a focus on the impact of social media addiction. Social media plays a dual role in providing vaccine information, with overexposure and misinformation contributing to hesitancy. Mistrust in institutions and scepticism towards vaccine benefit and commercial interests are recurring issues tied to social media. Targeting popular platforms like Instagram, WhatsApp, TikTok, Facebook and Snapchat for tailored vaccination campaigns is recommended to promote vaccine acceptance among nursing students.

## Funding

This study was funded by Sigma International—Sigma Global Nursing Research Grant and Northumbria University.

## Disclosure

Statistical data: The authors have checked to make sure that our submission conforms as applicable to the Journal's statistical guidelines. There is also a statistician on the author team (Goran Erfani). The authors affirm that the methods used in the data analyses are suitably applied to their data within their study design and context, and the statistical findings have been implemented and interpreted correctly. The authors agree to take responsibility for ensuring that the choice of statistical approach is appropriate and is conducted and interpreted correctly as a condition to submit to the Journal.

## Ethics Statement

Ethical approval was granted by Northumbria University Ethics Committee (ref: 2948 27/02/23) and all participants provided informed consent before participating in this study.

## Conflicts of Interest

The authors declare no conflicts of interest.

## Data Availability

The data that support the findings of this study are available on request from the corresponding author. The data are not publicly available due to privacy or ethical restrictions.
